# Emotion Processing, Reappraisal, and Craving in Alcohol Dependence: A Functional Magnetic Resonance Imaging Study

**DOI:** 10.3389/fpsyt.2019.00227

**Published:** 2019-04-09

**Authors:** Jochem M. Jansen, Odile A. van den Heuvel, Ysbrand D. van der Werf, Stella J. de Wit, Dick J. Veltman, Wim van den Brink, Anna E. Goudriaan

**Affiliations:** ^1^Institute for Addiction Research, Department of Psychiatry, Amsterdam UMC, University of Amsterdam, Amsterdam, Netherlands; ^2^Institute for Criminal Law & Criminology, Faculty of Law, Leiden University, Leiden, Netherlands; ^3^Department of Psychiatry, Amsterdam UMC, Vrije Universiteit Amsterdam, Amsterdam, Netherlands; ^4^Department of Anatomy & Neurosciences, Amsterdam Neuroscience, Amsterdam UMC, Vrije Universiteit Amsterdam, Amsterdam, Netherlands; ^5^Arkin Mental Health, Amsterdam, Netherlands

**Keywords:** alcohol dependence, emotion reappraisal, craving, functional magnetic resonance imaging, emotion regulation

## Abstract

Alcohol dependence has long been related to impaired emotion regulation—including reappraisal—but little is known about the performance and associated neural activity of alcohol-dependent patients (ADPs) on an emotion reappraisal task. This study, therefore, compares reappraisal of negative, positive, neutral, and alcohol-related images at a behavioral and neural level between ADPs and healthy controls (HCs).

Thirty-nine ADPs and 39 age-, gender-, and education-matched HCs performed an emotion reappraisal task during functional magnetic resonance imaging (fMRI), and craving was measured before and after the reappraisal task. During the emotion reappraisal task, participants were instructed to either attend or reappraise positive, negative, neutral, or alcohol-related images, and to indicate their experienced emotion on a visual analogue scale (VAS).

Both ADPs and HCs completed the emotion reappraisal task successfully, showing significant differences in self-reported experienced emotion after attending versus reappraising visual stimuli and in brain activity in emotion processing/reappraisal relevant areas. ADPs were not impaired in cognitive reappraisal at a behavioral or neural level relative to HCs, nor did ADPs indicate any difference in self-reported emotion while attending emotional images. However, ADPs were different from HC in emotion processing: ADPs revealed a blunted response in the (posterior) insula, precuneus, operculum, and superior temporal gyrus while attending emotional images compared neutral images compared to HCs, and in ADPs, higher baseline craving levels were associated with a less blunted response to alcohol-related images than in HCs. These results reveal that ADPs do not show impaired reappraisal abilities when *instructed*, although future studies should assess voluntary reappraisal abilities in alcohol-dependent patients.

**Clinical Trial Registration:** www.ClinicalTrials.gov, identifier NCT02557815.

## Introduction

The ability to manage emotional information is central to our daily functioning, and adequately managing emotions is achieved through various emotion regulation strategies, including attention shifting and cognitive reappraisal, the process of moderating the emotional impact of a certain thought or stimulus through cognitive reinterpretation (1, 2). Neuroimaging studies using reappraisal tasks in healthy controls (HCs) indicate that the prefrontal cortex, including the dorsolateral prefrontal cortex (dlPFC), is vital for the *regulation* of emotions, whereas the limbic system—including the amygdala and insula—is important for the initial *processing* of emotions (1–4). Other brain areas related to reappraisal include the dorsomedial prefrontal cortex, superior temporal gyrus, dorsal part of the anterior cingulate cortex (ACC), superior parietal lobule, and inferior frontal gyrus (4). These brain regions are part of cognitive–linguistic control networks, associated with effortful (i.e., explicitly applied) reappraisal by cognitively reframing the affective meaning of a negative stimulus in more neutral terms (2, 5).

Impairments in reappraisal are supposed to be related to the development, persistence, and severity of substance dependence (6). Previous studies have indicated that difficulties in coping with negative affect is one of the most prominent clinical factors in substance dependence (7). The induction of negative affect may increase the urge to drink (8, 9), although a recent study failed to show such a relationship between emotional state and craving for alcohol-dependent individuals (10). Impaired emotional reappraisal also predicts negative outcomes, including relapse, in substance use disorder (SUD) patients (11, 12). A recent study showed impaired emotional reappraisal (ER) in Internet gaming disorder patients compared to drug-naïve controls, suggesting that impaired emotion reappraisal might precede neurotoxic effects of alcohol or other substances (13). Together, these studies indicate that emotional reappraisal is central in the etiology of alcohol dependence.

The results from the aforementioned studies on emotional reappraisal in substance dependence are further corroborated by a recent review on the neural circuitry of impaired reappraisal in patients with SUDs compared with HCs. This review showed decreased recruitment of the ACC, dlPFC, and ventromedial prefrontal cortex (vmPFC) during reappraisal, but no differences in amygdala or insular functioning (14). The review therefore concludes that emotion regulation disturbances in substance dependence are related to impaired prefrontal functioning and not to excessive reactivity to emotional stimuli.

Most studies reviewed by Wilcox et al. (14) did not apply an (explicit) reappraisal task, but included emotion reactivity, implicit reappraisal, or behavioral control tasks, and therefore little is known about the neural circuitry of explicit reappraisal in substance use disorders in general and more specifically in alcohol-dependent patients (ADPs). The available studies into emotion regulation in alcohol dependence reveal that impaired emotion regulation is associated with increased craving levels, especially for ADPs who experience increased negative and decreased positive affect (15). Furthermore, interview data demonstrate that ADPs show reduced use of effortful cognitive emotion regulation and tend to apply less beneficial emotion regulation strategies like response modulation and attentional deployment strategies in daily life (16). ADPs also report problems with the identification and regulation of emotions (17), which are linked to the duration of the last heavy drinking episode, as well as higher drinking rates at 1-year follow-up (18). It is currently not clear, however, whether ADPs perform differently on an explicit cognitive reappraisal task and whether related brain activity is different.

The review by Wilcox et al. (14) further concludes that no differences were found in the limbic system, indicating that impaired reappraisal may originate from prefrontal impairments rather than from an excessive response to emotional stimuli. Some studies even point toward lower limbic responsivity to emotional stimuli in SUDs (19, 20), which fits with the findings regarding reduced salience of natural reinforcing stimuli, relative to addiction-relevant stimuli (21).

The current study is the first to assess differences in cognitive reappraisal abilities between ADPs and HCs at the behavioral and the neural level. We hypothesize that ADPs show decreased reappraisal abilities compared to HCs, indicated by self-report scores on a visual analogue scale (state), an emotion regulation questionnaire (trait). Reduced ER-related brain activity in areas such as the dlPFC and ACC is mainly expected for the reappraisal of negative emotion, which has been implicated in substance dependence (22), whereas the ER of alcohol-related images may either result in lower activity [in line with findings from Wilcox et al. (14)] or higher activity (due to increased cognitive load associated with higher salience of these images). We furthermore hypothesize no differences in brain activity during emotional processing of negative and positive images, but greater activations to alcohol-related images in ADPs compared to HCs. Finally, we expect craving levels to increase due to the emotion reappraisal task, and that craving is negatively related to cognitive reappraisal abilities.

## Methods and Materials

### Participants

A total of 39 ADPs (26 males) and 39 HCs (22 males) were included in this between-subjects study and were matched on (mean) age, sex, and education. ADPs were sober for at least 3 weeks and were recruited from addiction treatment centers in the larger city area of Amsterdam, the Netherlands. Sobriety was confirmed with a urine test in the research lab on the test days. None of the participants were active users of psychoactive medication, cannabis, opioids, or stimulants. HCs were recruited through Internet and social media advertisements. All participants were screened for MRI suitability. All subjects were screened (and if positive excluded) for the presence or history of psychiatric disorders, including substance abuse or dependence, using the Composite International Diagnostic Interview (CIDI) (23). The study was approved by the local Medical Ethical Commission of the Academic Medical Center of the University of Amsterdam and participants signed the informed consent form, consistent with the Declaration of Helsinki, before participating in the study. Participants were remunerated for their participation.

### Questionnaires

In addition to the CIDI interview, the Alcohol Use Disorder Identification Test (AUDIT) (24), Beck’s Depression Inventory (BDI) (25), Beck’s Anxiety Inventory (BAI) (26), the Toronto Alexithymia Scale-20 (TAS-20) (27), and the Emotion Regulation Questionnaire (ERQ) (28) were administered to assess levels of depression, anxiety, alexithymia, and emotion regulation, respectively. Finally, craving was assessed with the Alcohol Urge Questionnaire (AUQ) (29) before and after the performance of the emotion reappraisal task.

### Emotion Reappraisal Task

Participants viewed 18 negative (e.g., vicious dog, plane crash), 18 positive (e.g., cute puppies, beautiful landscape), 18 neutral (e.g., people at work, neutral landscape), and 18 alcohol-related images (e.g., glass of beer, bottles of wine) on a screen using a mirror attached to the head coil. The negative, positive, and neutral images used in this task were selected from the International Affective Image Set (IAPS) (30). Negative images had a low valence (≤4.0) and high arousal (≥6.0), whereas neutral images had a mildly positive valence (4.5 < *x* <7.0) and low arousal (2.0 < *x* < 4.2) and positive images had high valence (≥7.0) and arousal (≥5.0), based on the original IAPS scores. The alcohol-related images were selected from Vollstädt-Klein et al. (31) and supplemented by alcohol-related images of popular Dutch alcoholic beverages. All alcohol-related images were separately validated in an independent sample for valence (3.0 < *x* < 6.0) and arousal (2.0 < *x* < 4.0).

**Figure 1:**
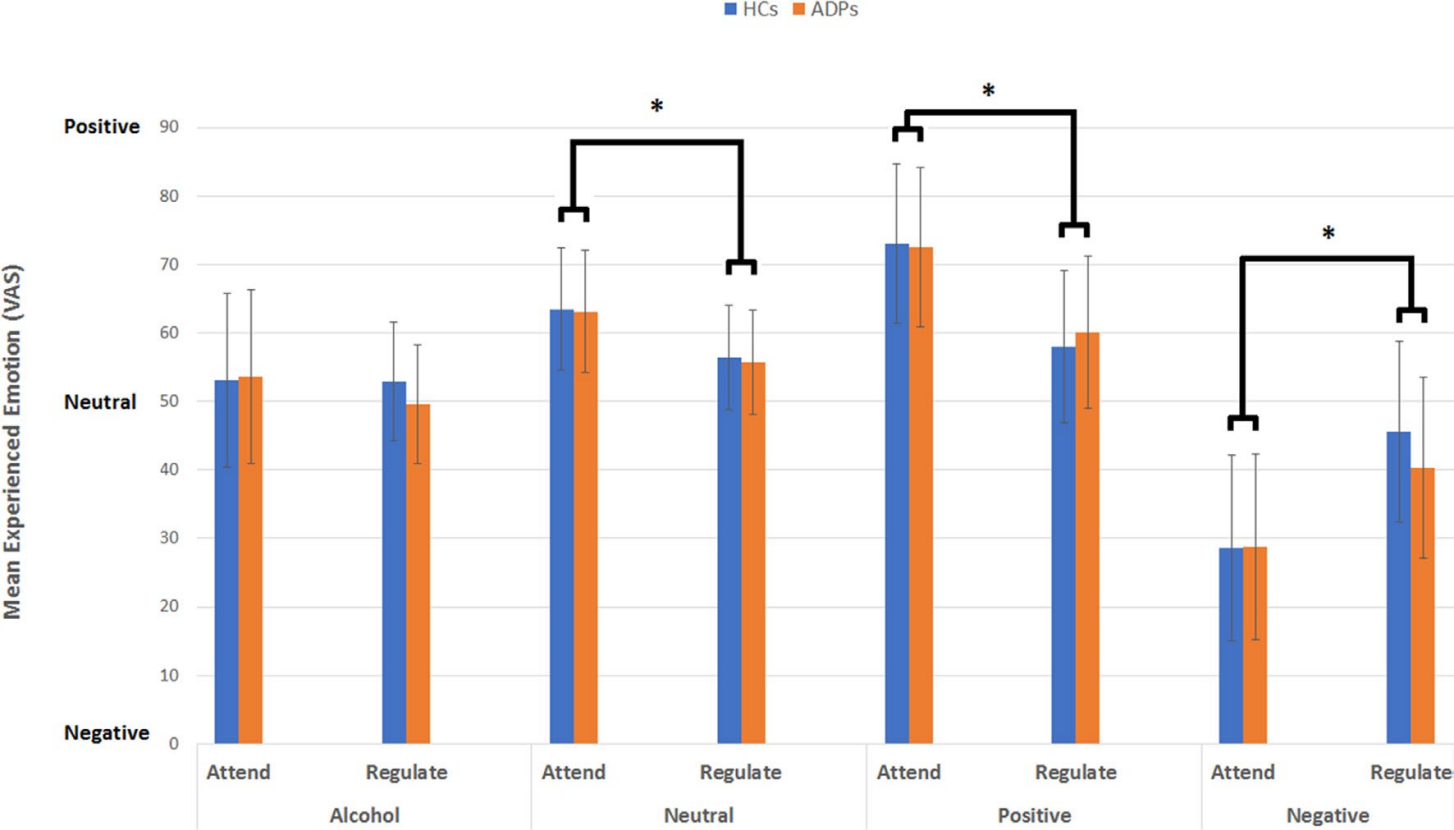
This figure reveals the mean experienced emotion (VAS) per emotion type, instruction, and participant group. Analysis reveals no effect of participant group, but a significant interaction between emotion type and instruction for alcohol-related, neutral, positive, and negative images. Error bars reflect the standard deviation.

The images were paired with one of two different instructions: “attend” and “reappraise.” In the attend instruction, participants were told to view and identify themselves with the situation in the image (e.g., “how would you feel in this situation”). In the reappraise condition, participants were told to reappraise their emotions related to these images in such a way that the negative feelings were reduced (e.g., “imagine a less negative outcome or interpretation”). Images were presented in 24 blocks of three images of the same emotion type (negative, positive, neutral, alcohol) with the same instruction (attend, reappraise) and presented in a pseudo-randomized order (see **Figure 1**).

After each image, for both instructions (attend and reappraise), a visual analogue scale (VAS) ranging from 0 to 100 was presented and participants had to rate their emotional state (“How do you feel?” where 0 is very negative, 50 is neutral, and 100 is very positive) by moving a bar to the right or left by pressing a button box multiple times. This moving bar was set in the middle (representing a neutral value of 50) and the range of emotions was indicated by previously validated self-assessment manikins depicting valence (32). Prior to scanning, the assessment was explained and practiced outside the scanner using example stimuli (not used in the experiments) for approximately 5 min. The reappraisal task itself took approximately 25 min.

### Analysis

#### Behavioral Analysis

Data were prepared for analysis by winsorizing extreme values for experienced emotion (mean VAS per condition) and craving (AUQ pre- and post-scores), by replacing values below the 5th and above the 95th percentile by the 5th or 95th percentile, respectively, and by confirming that experienced emotion was normally distributed.

In order to assess effects of emotion type, instruction, and participant group on experienced emotion, a general linear model (GLM) Univariate ANOVA was performed, including experienced emotion (mean VAS) as the dependent variable, and instruction (attend, reappraise), emotion type (alcohol, neutral, positive, negative) and participant group (ADP, HC) as fixed factors. Significant interactions were followed up by Bonferroni-corrected simple effects analyses. Independent sample *t* tests were performed to assess whether gender influenced experienced emotion per condition with results considered significant at a Bonferroni-corrected *p* = . 006 (0.05/8).

The AUQ was administered before (pre) and after (post) the reappraisal task. Due to the many mistakes that were made in the second and seventh question—which are reverse coded and were misinterpreted—these were excluded from the analysis. Both pre and post scores were positively skewed and therefore a log(*x* + 1) transformation was applied. A repeated-measures ANOVA was performed including AUQ scores as the dependent variable, time (pre/post) as the within-subjects factor and participant group as the between-group factor. Finally, the increases in craving levels (post- minus pre-AUQ scores) were correlated to the means of experienced emotion per emotion type and instruction.

##### Functional Magnetic Resonance Imaging

###### Data Acquisition

MRI scanning was performed on a Philips Achieva 3T scanner at the Spinoza Imaging Centre, Amsterdam, the Netherlands. Functional MRI [echo time (TE) = 27.63 ms; repetition time (TR) = 2000 ms; field of view (FOV) = 240 × 240 mm, 37 3-mm slices, 0.3-mm slice gap; 80 × 80 matrix; flip angle = 76.1°] was performed to acquire blood oxygenation level-dependent (BOLD) signals using single-shot multi-echo (33) T2*-weighted echo planar imaging (EPI’s). These T2-weighted flow-compensated 8 spin-echo anatomical images were oriented axially along the anterior commissure to the posterior commissure (AC–PC) line. During the baseline session, a T1-weighted 3D data set was obtained for anatomical reference; TR = 8.196 ms, TE = 3.73 ms, field of view (FOV) = 140 × 188 × 220 mm, matrix 240 × 187, flip angle = 8°, slice thickness = 1 mm, number of slices = 220.

###### Preprocessing and First-Level Analysis

Preprocessing was performed with SPM8 (Wellcome Trust Centre for Neuroimaging, London, United Kingdom) in MATLAB (version 2012b) and included realignment to the first image, slice timing correction to the middle (18th) slice, co-registration of the anatomical T1 of the subject to the mean functional scan, and warping of this co-registered T1 to standard space. Next, the volumes were normalized to the Montreal Neurological Institute (MNI) template and smoothed with a 7-mm Gaussian kernel in order to increase signal-to-noise ratio. To account for low-frequency drifts, a high-pass filter (128 Hz) was applied. Three subjects (two ADP and one HC) were removed due to the low quality of the fMRI data (e.g., scanner artifacts).

In the first-level model regressors of no interest were Instruction and VAS scoring. Instruction was modeled with a boxcar of 3 s, whereas VAS scoring was modeled with a boxcar for the true duration of the scoring process since this was self-paced. The eight regressors of interest included the onsets of the negative, positive, neutral, and alcohol-related images in either attending or reappraising condition, which were modeled as boxcars (duration, 5 s) and convolved with a hemodynamic response function, in the first-level, single-subject, fixed-effects analysis. First-level contrasts for reappraisal [reappraise > attend] were computed per emotion condition (negative, positive, alcohol, and neutral). For emotion processing, separate contrasts were created for attending emotional images (alcohol, positive, or negative) versus neutral images [attend emotion (positive, negative, alcohol) > attend neutral].

###### Functional Magnetic Resonance Imaging Data Analysis

Separate second-level fMRI analyses were performed for the attend and reappraise conditions. For the attend condition, a 2 × 3 ANOVA was conducted in SPM12, including the [attend emotion > attend neutral] contrast per emotion, in order to assess the interaction between group (ADP, HC) and emotion (alcohol, positive, negative) as well as main effects of group, emotion, and condition. For the reappraise condition, a 2 × 4 ANOVA conducted in SPM12, including the [reappraise > attend] contrasts per emotion, in order to assess the interaction between group (ADP, HC) and emotion (alcohol, neutral, positive, negative) as well as main effects of group, emotion, and condition.

First, the main effects of instruction (attend, reappraise) during the emotion reappraisal task are discussed in order to confirm that the emotion reappraisal task was completed successfully. Then, the group by emotion interactions, as well as the main effects for group and emotion will be discussed. Results are reported at a whole-brain *p* < 0.05 FWE-corrected threshold; furthermore, amygdala Region of Interest (ROI) analyses (based on the BrainMap database) were performed for the attend condition.

In order to assess whether craving is positively correlated to higher brain responsivity during emotion processing and negatively correlated to brain activity during emotion reappraisal, any significant differences in brain activity between ADPs and HCs were followed up by a Pearson correlation analysis, including the extracted individual *b* values from the peak-voxel coordinate, craving levels before the emotion reappraisal task, and the increase in craving levels due to the emotion reappraisal task.

## Results

### Demographics

ADPs and HCs were successfully matched on age, gender, and years of education. However, ADPs reported significantly higher levels of depression (BDI), anxiety (BAI), and alexithymia (TAS-20). Analyses were not corrected for these differences, because depression, anxiety, and alexithymia levels are well known to be elevated in alcohol dependence (34–37). There were no group differences in the ERQ scores (**Table 1**).

**Table 1:** Sample characteristics. This table shows the results for the analyses of the sample characteristics. Values are denoted as mean (standard deviation). Total number of participants per comparison may vary due to a small number of missing values. SD, standard deviation; AUDIT, Alcohol Use Disorders Identification Test; TAS, Toronto Alexithymia Scale; DIDF, difficulties identifying and describing feelings; EOT, externally oriented thinking; ERQ, emotion regulation questionnaire. ERQ Reappraisal and Suppression are subscales of the ERQ.

	Possible range (min–max)	Mean ADP (SD) n = 39	Mean HC (SD) n = 39	Significance
Age		41.64 (8.63)	44.05 (10.52)	*t*(1,76) = 1.11, *p* = .27
Years of education		15.31 (3.05)	15.35 (2.98)	*t*(1,71) = .64, *p* = .95
Gender		*M* = 26	*M* = 22	χ^2^(1,78) = .87, *p* = .35
AUDIT	0–41	22.11 (10.51)	4.17 (2.51)	*t*(1,71) = 9.97, *p* < 0.001
TAS-20 total	20–100	51.43 (10.83)	43.06 (8.62)	*t*(1,67) = 3.54, *p* = 0.001
TAS-20 DIDF	12–60	31.83 (8.16)	24.86 (7.20)	*t*(1,68) = 3.79, *p* < 0.001
TAS-20 EOT	8–40	11.97 (3.30)	11.36 (2.73)	*t*(1,71) = .86, *p *= .39
ERQ total	7–70	37.81 (7.95)	36.32 (8.56)	*t*(1,71) = .77, *p* = .45
ERQ Reappraisal	6–42	20.22 (5.87)	19.00 (7.80)	*t*(1,72) = .76, *p* = .45
ERQ Suppression	4–28	17.72 (5.01)	17.32 (5.10)	*t*(1,72) = .35, *p* = .73
Beck Depression Inventory	0–63	10.84 (9.58)	4.27 (6.28)	*t*(1,72) = 3.39, *p* = .001
Beck Anxiety Inventory	21–84	30.40 (8.73)	24.18 (4.56)	*t*(1,74) = 3.89, *p* < .001

### Task Effects and Group Difference (Behavior)

#### Negative, Positive, Alcohol-Related, and Neutral Images

The three-way repeated-measures ANOVA with experienced emotion (mean VAS per condition) as the dependent variable, emotion type (negative, positive, neutral, alcohol related) and instruction (attend, reappraise) as within-subject factors, and group (ADP, HC) as between-subject factors did not reveal a significant three-way interaction [*F*(3,624) = 1.06, *p* = .36, *d* = .14]. Two-way interactions between participant group and instruction [*F*(1,624) = .53, *p* = .47, *d* = .06] or participant group and emotion type [*F*(3,624) = .19, *p* = .90, *d* = .06] also did not reveal any significant effect.

Results did reveal a significant interaction between emotion type and instruction [*F*(3,624) = 39.11, *p* < 0.001, *d* = .88], indicating that experienced emotion varied between emotion type and instruction. Simple effects analysis for this interaction revealed a significant difference between attending and reappraising neutral [mean difference = 7.66; *F*(1,624) = 17.06, *p* < 0.001, *d* = .33], positive [mean difference = 13.52; F(1,624) = 53.24, *p* < 0.001, *d* = .59], and negative images [mean difference = −13.46; *F*(1,624) = 52.79, *p* < 0.001, *d* = .59]. There was no difference between attending and reappraising alcohol-related images [mean difference = 2.59; *F*(1,624) = 1.96, *p* = .16, *d* = .11]. These results indicate that attending neutral [mean = 64.08, SD = 9.84] and positive (mean = 71.10, SD = 11.31) images resulted in the experience of positive emotions, which were reduced during reappraise condition for both neutral (mean = 56.43, SD = 7.58) and positive images (mean = 59.58, SD = 11.41). Attending negative images on the other hand resulted in the experience of negative emotion (mean = 28.13, SD = 13.04), which were reduced (i.e., less negative) in the reappraise condition (mean = 41.60, SD = 12.77; see **Figure 1**).

Independent-sample *t* tests assessing whether gender influenced experienced emotion during attending revealed no difference in positive, negative, neutral, or alcohol-related images, and also no differences during regulating positive, negative, or neutral images (all *p* > 0.006). However, female participants experienced more positive emotions during regulating alcohol-related images than male participants [mean = 56.15 (SD = 12.85) vs. mean = 47.27 (SD = 13.01), respectively; *t*(71) = 2.85, *p* = .006].

#### Craving

**Figure 2:**
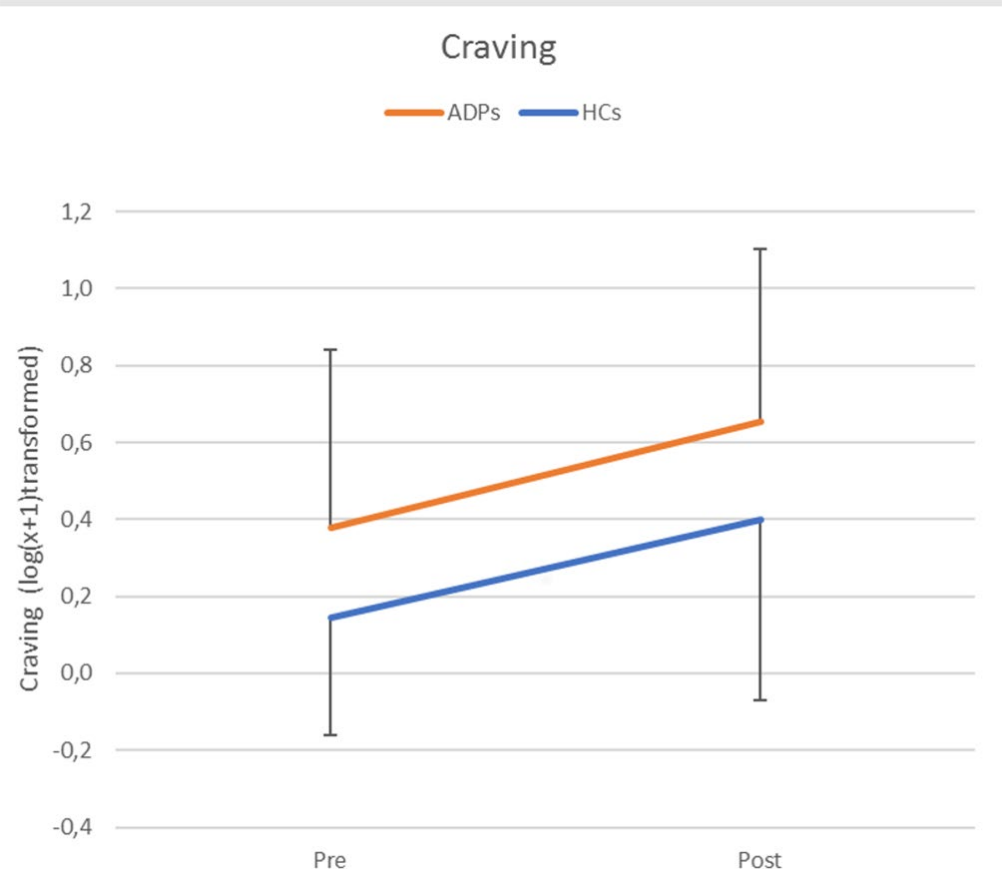
Craving levels per group and time point [pre/post-emotion reappraisal task (ERT)]. Error bars reflect standard deviations. Craving levels were log(*x* + 1) transformed and refer to self-reported Alcohol Urge Questionnaire (AUQ) scores.

The repeated-measures ANOVA assessing craving levels revealed no significant interaction between time (pre/post) and participant group [ADP/HC; *F*(1,71) = .06, *p* = .81, *d* = 0.06], but significant main effects for time [*F*(1,71) = 29.42, *p* < 0.001, *d* = 1.29] and group [*F*(1,71) = 7.57, *p* < 0.01, *d* = .65]. These results indicated that the emotion reappraisal task significantly increased craving levels in both ADPs and HCs to an equal extent, but that craving levels in ADPs were overall higher (see **Figure 2**). The increase in craving (post/pre) did not correlate with experienced emotion per instruction and emotion type (all *p* values >0.1) in either ADPs, HCs, or over all subjects.

### Functional Magnetic Resonance Imaging Results

#### Main Task Effects

**Figure 3:**
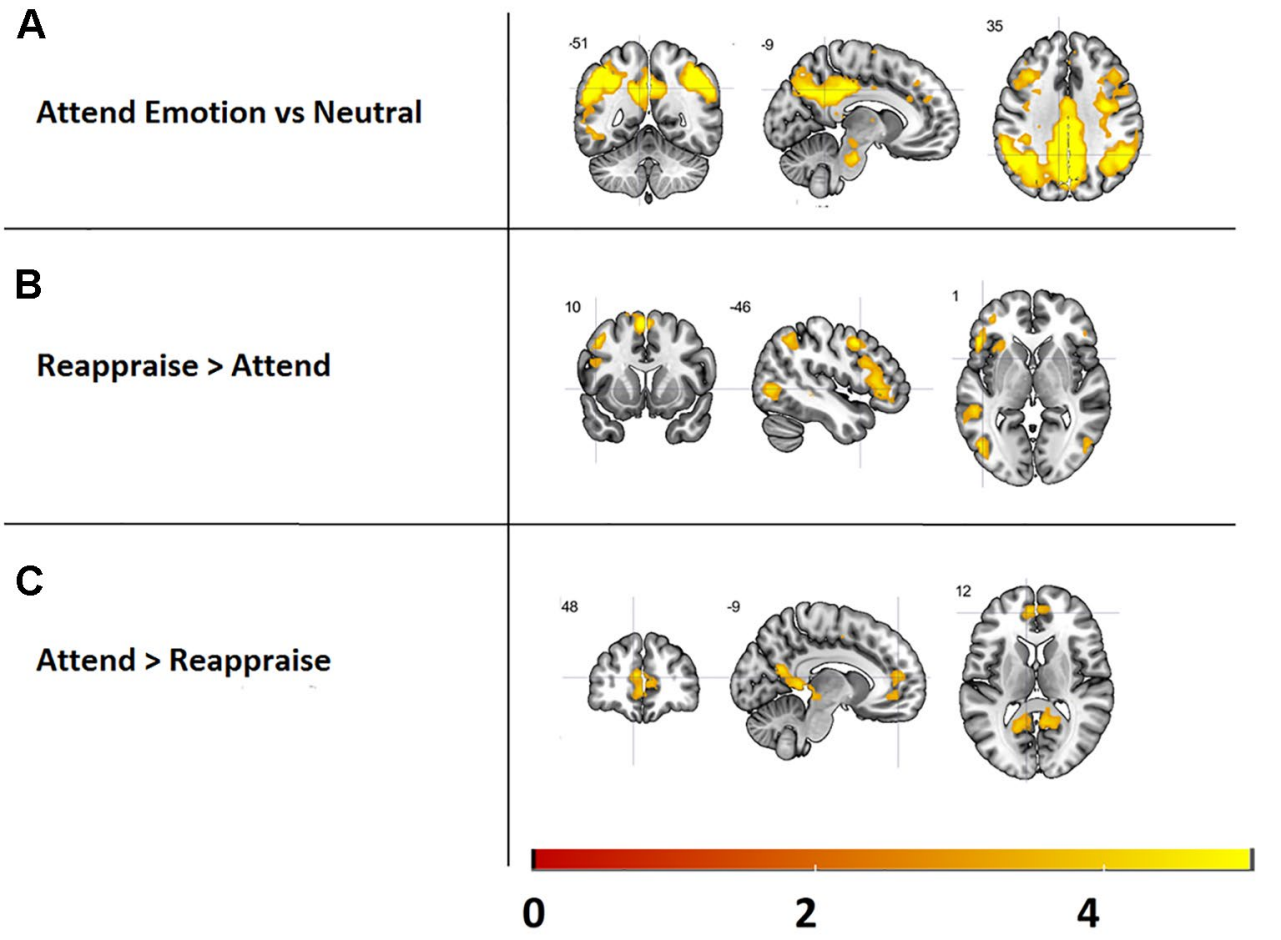
Main effects of the emotion reappraisal task, presented at a threshold of *k* > 5, *p* < 0.001. Top: Brain areas activated while attending emotional images versus neutral images. Middle: activated brain areas during reappraising vs. attending images. Bottom: regions more activated during attending images vs. reappraising images.

In order to check the experimental manipulation of the emotion reappraisal task, the main effects of task, i.e., attend [attend emotion > attend neutral] and reappraise [reappraise > attend], were assessed for all emotions and both groups combined (see **Figure 3** and **Supplementary Table 1**). Results revealed that attending emotional images (versus neutral images) increased activity in the visual stream and posterior parietal cortex as well as the precentral gyrus. Our ROI analysis revealed no significant activations in the amygdala.

Reappraising images (versus attending) resulted in increased activation in several cortical structures previously implicated in emotion reappraisal, including the interior frontal gyrus, supplementary motor cortex, and middle frontal gyrus (see **Figure 3** and **Supplementary Table 1**). Furthermore, activity in the visual stream and the medial segment of the superior frontal gyrus was significantly higher during the attend relative to the reappraise condition (see **Figure 3** and **Supplementary Table 1**).

### Group Differences in Brain Activation

#### Emotion Processing

**Figure 4:**
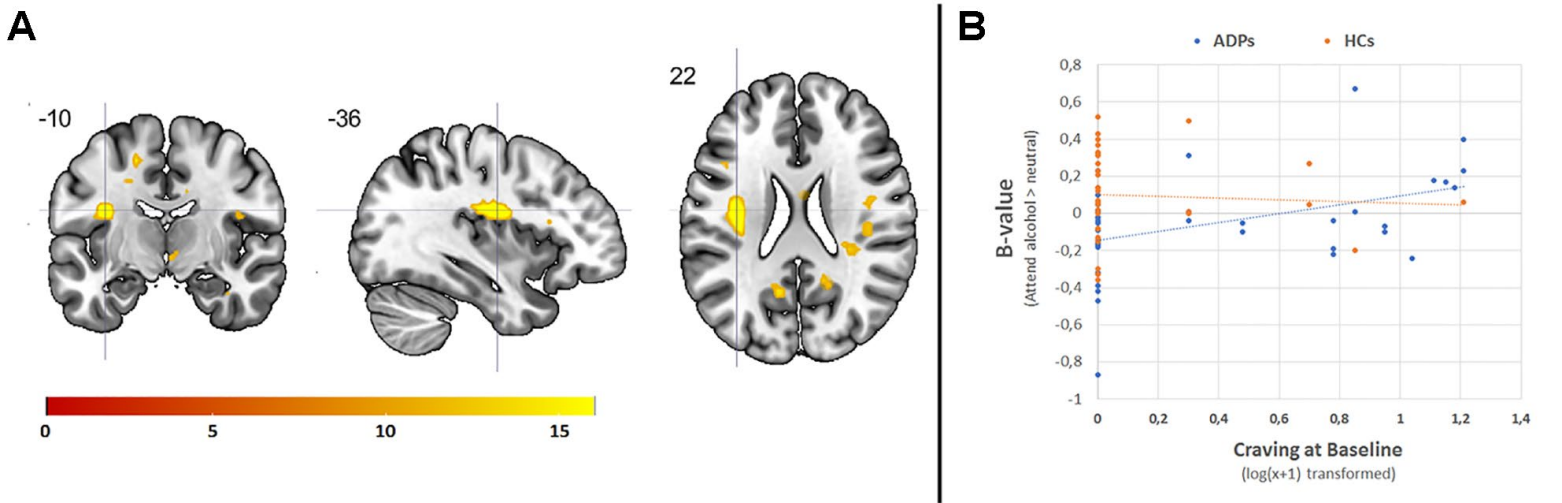
**(A)** Graphical presentation of the increased activity in healthy controls (HCs) compared to alcohol-dependent patients (ADPs), during emotion processing (attending negative, positive, and alcohol-related images vs. neutral). Crosshair is pointed at the peak voxel in the posterior insula. **(B)** The correlation between the posterior insula and baseline craving levels for the [attend alcohol > neutral] contrast. The threshold for visualization of the results is set at *k* > 5, *p* < 0.001 uncorrected.

The 3 × 2 ANOVA including the [attend emotion > attend neutral] contrasts per emotion (alcohol, positive, negative) and group (ADP, HC) revealed no significant interaction. The main effect of group showed that HCs have higher activity in the bilateral central operculum, precuneus, and superior temporal gyrus during appraising stimuli (see **Table 2** and **Figure 4A**). Furthermore, there was a significant main effect of emotion within the visual stream, but since these effects are not of main interest, they are reported in **Supplementary Information 2**.

**Table 2:** Main effect of participant group for attending emotional vs. neutral images. *T*, *t* value; *K*, cluster size in voxels; *x*, *y*, *z* are coordinates.

Brain area (attend emotion > neutral)	L/R	*T*	*K*	*x*	*y*	*z*	*p* value (FWE-corrected)
HC > ADP							
Posterior Insula	Left	5.22	70	−36	−10	22	<.001
Parietal Operculum	Right	5.06	100	33	−34	19	0.01
Precuneus	Right	5.00	37	15	−55	28	.013
Central Operculum	Right	4.73	25	42	−7	19	.039
Superior Temporal Gyrus	Right	4.67	18	−21	−7	40	.048
ADP > HC							
n.a.							

*Post hoc* correlations between the posterior insula (peak voxel), which was significantly more activated during emotion processing (attend emotion > neutral), and both baseline craving levels and the increase in craving levels due to the emotion reappraisal task indicate a significant correlation for baseline craving levels with the posterior insula only for the APDs [*r*(37) = .36, *p* = .03] and not for the HCs [*r*(33) = −.05, *p* = .76]. This correlation seems to be related to the response to alcohol-related images in ADPs [*r*(37) = .43, *p* < .01, see **Figure 4B**], rather than the response to positive [*r*(37) = .28, *p* = .09] or negative images [*r*(37) = .21, *p* = .21].

#### Emotion Reappraisal

The 4 × 2 ANOVA for including the [reappraise > attend] contrasts per emotion (alcohol, neutral, positive, negative) and group (ADP, HC) revealed no significant interaction or any main effect of group. There was, however, a significant main effect of emotion, indicating a difference in neural response between the neutral, alcohol, positive, and negative images in the visual stream. Since these results are not of main interest, they are reported in **Supplementary Information 2**.

## Discussion

This study investigated differences in emotion processing and reappraisal between alcohol-dependent patients (ADPs) and healthy controls (HCs) at the behavioral and the neural level. The emotion reappraisal task was completed successfully as indicated by increased experienced emotion in the attend condition and successful regulation of these emotions in the reappraisal condition. Our results do not show that ADPs have impaired emotion reappraisal based on this paradigm and the emotion regulation questionnaire, nor do they show any difference between ADPs and HCs in neural recruitment during cognitive reappraisal. However, our results do show a reduced neural response to emotional images (in comparison to neutral images) in ADPs versus HCs in the central operculum, precuneus, and superior temporal gyrus. Furthermore, self-reported craving levels increased from pre- to post-testing similarly in both groups, although overall craving levels were significantly higher in ADPs. Finally, self-reported baseline craving levels were correlated to higher neural reactivity to attending alcohol-related images in the ADP group.

The abovementioned results mostly do not correspond with our hypotheses, since we expected reduced emotion regulation ability and decreased associated brain activity in ADPs compared to HCs. These hypotheses were based on a recent review, which indicated reduced emotion regulation abilities and brain function in substance use disorders (14). However, the studies included in this review were mostly studies on emotional reactivity, implicit reappraisal, or behavioral control tasks and are therefore different from our explicit reappraisal paradigm. This may well be a major explanation for our (lack of expected) results, since explicit emotion regulation requires conscious effort, monitoring, and insight, whereas implicit emotion regulation is more automatic. Previous studies also show that ADPs make less use of these effortful emotion regulation strategies in daily life (16). Our results may differ from these previous studies because our participants were actively instructed to regulate their emotions by applying effortful cognitive reappraisal strategies. The fact that we do not show impaired emotion regulation abilities or differences in related brain function when *instructed* to apply these strategies may point to impairments in the selection of the appropriate reappraisal strategy rather than the ability itself. Of note, the ERQ also did not reveal any differences in emotion reappraisal or suppression between ADPs and HCs. This result is surprising, since the ADP group did self-report higher levels of anxiety and depression. Nevertheless, limited availability and access to emotion regulation strategies has been suggested and found by Khosravani et al. (15) and supports the aforementioned explanation. These results may imply that treatment should focus on selecting the right reappraisal strategy, rather than on reappraisal abilities.

In line with our hypothesis, we show that ADPs and HCs score their experienced emotion (using VAS scores) equally during the attend condition for all emotion types. However, ADPs do show significantly lower brain activity during attending the stimuli in several brain areas, including the posterior insula, central operculum, precuneus, and superior temporal gyrus. These findings are in line with previous studies suggesting a blunted neural response to emotional images in APDs (20) and marijuana smokers (19), participants with excess weight (38), and with studies hypothesizing a blunted response to non-addiction-relevant emotional stimuli (21).

In contrast with our hypothesis, the reappraisal task induced craving equally in both ADPs and HCs. This may be explained by a mismatch between specific preferences from the individual ADPs (e.g., someone who only drinks beer) and the diversity of alcohol-related images that were presented (beer, wine, liquor, bar, supermarket), which may have dampened the craving inducing effect. Future studies should consider a personalized approach, matching the presented images to the subject’s specific preference.

Comparing our data to data from the Dutch national monitoring system for drug- and alcohol-dependent patients (39), our ADP group was slightly younger (41 years vs. 46 years), but gender distribution was comparable (67% vs. 72% male). Our ADP participants were in treatment for alcohol dependence but were medication free since the use of psychoactive medication was an exclusion criterion. This is atypical for most treatment-seeking alcohol-dependent patients who are often prescribed anti-craving medication. It is possible that patients who are not prescribed any medication (e.g., our participants) experience less craving compared to ADPs who are prescribed medication since severe craving can be an indication to prescribe medication. Possibly the ADPs included in this study experienced less craving than ADPs who are prescribed psychoactive medication, which may explain the similar effects of the emotion regulation task on craving levels for ADPs and HCs in this study. Our *post hoc* correlations in APDs are in line with this explanation, since they reveal that higher baseline craving levels are associated with more activity within the posterior insula while attending alcohol-related images. In other words, ADPs who experience higher baseline craving levels have a stronger neural response to alcohol-related images in a brain region that has previously been implicated in cue-induced craving in alcohol-dependent patients (22).

Together, these results suggest that ADPs show a blunted response to emotional images when compared to HC, but also that within the ADP group, higher craving levels are associated with a “less” blunted neural response to alcohol-related images. Previous studies indicate that reduced responsiveness to emotional cues could be caused by reduced salience of these cues in comparison to addiction-relevant cues (21) and these findings are in line with our results. Another explanation, which we could not confirm with the available data, is that a reduced neural reaction to emotional images may serve as an implicit protective mechanism. Since a higher response to emotional images has been linked to craving (22), reducing this response may lead to less craving. This explanation, however, is speculative and should be investigated further.

### Strengths and Limitations

The current study assessed emotion reappraisal as well as emotion processing in alcohol dependence through a comprehensive study, using both questionnaires, behavioral data, as well as fMRI. Despite the strengths of this study, we only studied one form of emotion regulation (reappraisal), and future studies should incorporate multiple emotion regulation strategies, including, e.g., voluntary emotion reappraisal, avoidance, or distraction. Although the reappraisal task induced craving in ADPs and HCs, it is not possible to clarify which images or conditions caused this effect because craving was measured only before and after the reappraisal task, and this may be an explanation why none of the conditions correlated with the increase in craving levels.

The lack of a clear distinction in emotional reappraisal between ADPs and HCs might be explained by insufficient emotional impact of the images that were used in the task. The IAPS database images may lack ecological validity, thus reducing the impact of these images and thus facilitating the emotion reappraisal process. On the other hand, using a comparable task (without the alcohol-related images), we were previously able to differentiate between HCs and patients with obsessive–compulsive disorder during emotion processing, but not during emotional reappraisal (40). Future studies should consider other ways of inducing emotions with higher ecological validity, including personalized scripts, personalized images, or the use of virtual reality. Additionally, future studies should consider incorporating measurements of personality disorders, including borderline personality disorder, that have previously been linked to impaired emotion reappraisal (41), but were not used in the current study.

## Conclusion

The current study showed neither impaired reappraisal of emotion in ADPs nor reappraisal-related differences in brain activity in ADPs compared to HCs. The results might have been influenced by some methodological limitations, although we did demonstrate a blunted neural response in ADPs while attending emotional (positive, negative, alcohol-related) images. Moreover, baseline craving levels were correlated to a less blunted neural response to alcohol-related images in ADPs. Together, these results may suggest a link between emotional reactivity and craving, and impaired natural emotion processing in alcohol dependence, whereas ADPs show unimpaired reappraisal abilities when explicitly *instructed*. Future studies should assess voluntary reappraisal abilities, more ecologically valid ways of inducing emotions, and compensatory mechanisms in ADPs to further understand the differences during natural (re)appraisal of emotional cues.

## Ethics Statement

This study was carried out in accordance with the recommendations of the Medical Ethical Commission of the Academic Medical Center of the University of Amsterdam with written informed consent from all subjects. All subjects gave written informed consent in accordance with the Declaration of Helsinki. The protocol was approved by the local Medical Ethical Commission of the Academic Medical Center of the University of Amsterdam.

## Author Contributions

All authors made a significant contribution to this article, including acquiring funding (AG and WB), study design (AG and WB), development of the emotion reappraisal task (SW, OH, DV, and YD), data acquisition (JJ), data analysis (JJ, DV, and SW), interpretation of results (JJ, AG, SW, OH, YD, WB, and DV) and contributions to this manuscript (JJ, AG, SW, OH, YD, WB, and DV).

## Funding

This research was partly funded by The European Foundation for Alcohol Research (ERAB); grant no. EA1027 to AG, WB, and DV; and by a VIDI grant (no: 91713354) from the Dutch Scientific Foundation to AG.

## Conflict of Interest Statement

The authors declare that the research was conducted in the absence of any commercial or financial relationships that could be construed as a potential conflict of interest.
