# Supplementary material for: Emotion Processing, Reappraisal, and Craving in Alcohol Dependence: A Functional Magnetic Resonance Imaging Study
Source: Front Psychiatry. 2019 Apr 9;10:227. doi: 10.3389/fpsyt.2019.00227 (PMC6465626; doi:10.3389/fpsyt.2019.00227)
Supplement: Supplementary file 1 [file Table_1.docx]

Supplementary Information 1: Main effect of instruction

| Brain Area  (attend emotion > neutral) | L/R | T | K | X | Y | Z | P-value (fwe-corrected) |
| --- | --- | --- | --- | --- | --- | --- | --- |
| Angular Gyrus  (posterior partietal cortex) | left | 7.25 | 3243 | -51 | -6  1 | 40 | <.001 |
| Angular Gyrus | right | 6.97 | 742 | 45 | -61 | 46 | <.001 |
| Precentral/Middle Frontal Gyrus | right | 27.39 | 621 | 36 | -4 | 34 | <.001 |
| Middle Frontal Gyrus | right | 5.46 | 26 | 30 | 56 | 7 | .002 |
| Middle Frontal Gyrus | left | 5.43 | 539 | -39 | 26 | 22 | .002 |
| Superior Frontal Gyrus | right | 5.20 | 196 | 6 | 35 | 46 | .006 |
| Anterior Insula | left | 5.13 | 94 | -42 | 14 | -11 | .008 |
| Thalamus | right | 4.90 | 87 | 15 | -7 | 7 | .02 |
| Brain stem | left | 4.77 | 121 | -9 | -22 | -29 | .033 |
| Occipital Fusiform Gyrus | right | 4.75 | 25 | 21 | -82 | -17 | .036 |
| Occipital Pole | right | 4.70 | 49 | 15 | -97 | 10 | .044 |
| Cerebellum | left | 4.66 | 77 | -24 | -73 | -20 | .05 |

Supplement table 1. Shows brain areas activated for attending emotional images versus neutral images at a p<0.05 whole brain FWE-corrected level. T = t-value, K = cluster size in voxels, x,y,z = coordinates.

| Brain Area  Reappraise > attend | L/R | T | K | X | Y | Z | P-value (fwe-corrected) |
| --- | --- | --- | --- | --- | --- | --- | --- |
| Supplementary Motor Cortex | left | 5.99 | 267 | -3 | 5 | 64 | <.001 |
| Inferior Frontal Gyrus | left | 5.57 | 706 | -51 | 26 | 4 | .001 |
| Supra Marginal Gyrus (posterior parietal cortex) | left | 5.54 | 354 | -54 | -49 | 46 | .001 |
| Middle Temporal gyrus | left | 4.96 | 233 | -43 | -37 | -2 | .014 |
| Attend > reappraise |  |  |  |  |  |  |  |
| Lingual Gyrus  (visual stream) | left | 6.44 | 1266 | -21 | -40 | -11 | <0.001 |
| Superior frontal gyrus  (medial segment) | left | 4.70 | 350 | -6 | 50 | 13 | .041 |

Supplement Table 2. This table shows the brain areas with increased (top) and decreased (bottom) activation during the reappraise (vs attend) condition at a whole brain p<.05 FWE-corrected level. T = t-value, K = cluster size in voxels, x,y,z = coordinates.

Supplementary Information 2: Main effect of Emotion

| Brain Area | Effect | L/R | F | K | X | Y | Z | P-value (fwe-corrected) |
| --- | --- | --- | --- | --- | --- | --- | --- | --- |
| Fusiform Gyrus | Pos<alc=neg | Left | 39.04 | 355 | -30 | -49 | -14 | <.001 |
| Inferior Occipital Gurys | Alc<pos=neg | Right | 33.49 | 52 | 54 | -67 | 4 | <.001 |
| Calcarine Cortex | Pos<alc=neg | Right | 30.55 | 304 | 6 | -88 | 1 | <.001 |
| Middle Occipital Gyrus | Pos>alc=neg | Left | 27.28 | 31 | -30 | -91 | 10 | <.001 |
| Middle Occipital Gyrus | Pos>alc=neg | Right | 19.65 | 32 | 36 | -85 | 13 | <.001 |
| Fusiform Gyrus | Alc<pos<neg | Left | 18.73 | 5 | -42 | -46 | -17 | <.001 |

Supplementary table 2.1. Main effect of emotion for the processing of emotion. F = f-value, K = cluster size in voxels, x,y,z = coordinates.

| Brain Area | Effect | L/R | F | K | X | Y | Z | P-value (fwe-corrected) |
| --- | --- | --- | --- | --- | --- | --- | --- | --- |
| Lingual Gyrus | Pos > (alc=neu) > Neg | Right | 19.69 | 105 | 12 | -76 | -5 | <.001 |
| Fusiform Gyrus | Alc < (Neu = neg) < Pos | Left | 13.27 | 9 | -33 | -43 | -17 | .002 |

Supplementary table 2.2. Main effect of emotion for the reappraisal of emotion. F = f value, K = cluster size in voxels, x,y,z = coordinates.
